# Diseases Costs and Impact of the Caring Role on Informal Carers of Children with Neuromuscular Disease

**DOI:** 10.3390/ijerph18062991

**Published:** 2021-03-15

**Authors:** Alicia Aurora Rodríguez, Óscar Martínez, Imanol Amayra, Juan Francisco López-Paz, Mohammad Al-Rashaida, Esther Lázaro, Patricia Caballero, Manuel Pérez, Sarah Berrocoso, Maitane García, Paula María Luna, Paula Pérez-Núñez, Nicole Passi

**Affiliations:** Neuro-e-Motion Research Team, Faculty of Psychology and Education, University of Deusto, Av. Universidades, 24, 48007 Bilbao, Spain; oscar.martinez@deusto.es (Ó.M.); imanol.amayra@deusto.es (I.A.); jlopez@deusto.es (J.F.L.-P.); mohammad.alrashaida@deusto.es (M.A.-R.); estherlazaro@deusto.es (E.L.); patricia.caballero@deusto.es (P.C.); manuel.perez@deusto.es (M.P.); sarah.berrocoso@deusto.es (S.B.); maitane.garciamartin@deusto.es (M.G.); paula.lunao@deusto.es (P.M.L.); pau.perez@opendeusto.es (P.P.-N.); nicole.passi@opendeusto.es (N.P.)

**Keywords:** neuromuscular disease, carers, informal costs, psychological health, quality of life

## Abstract

This study aims to evaluate the costs of informal care for children with neuromuscular disease and evaluate how physical and psychological health is associated with socio-demographic variables. A cross sectional design was used with a convenience sample of 110 carers that participated in this study. Participants were recruited from Spanish hospitals and rare diseases organizations. Economic costs and sociodemographic aspects were assessed using the economic costs questionnaire and the sociodemographic questionnaire. Physical and psychological health was evaluated using the CarerQol-7D, PHQ-15, Barthel Index, Zarit Overload Scale and Satisfaction with Life Scale. Carers of children with neuromuscular disease spent a large percentage of their annual income in physical therapy, psychological care and speech therapy. Informal costs differed according to the degree of dependency of the child. These were higher in those caregivers whose child under their care presented low functional independence. The loss of work productivity was related to marital status, use of professional services and the child’s dependency. Finally, carers who were female, single or separated and without a job showed worse physical and psychological health. The results highlighted that carers have to face a number of high costs because of the non-existence of social protection and due to the child’s diagnosis.

## 1. Background

Neuromuscular diseases (NMDs) are an heterogeneous group of diagnoses that are genetic and usually hereditary. Their worldwide prevalence ranges from 7.1 to 26.5 per 100,000 people [1]. NMDs most important characteristics include progressive loss of muscular strength, atrophy or hypertrophy, fatigue, myalgia, muscular rigidity and the degeneration of all the muscles and nerves that control them [2]. At an early age, they need specific care, which is fundamentally provided by their relatives who are usually women [3].

Caring for a child with an NMD can have a serious impact on the carer’s life, with chronic stress being one of the most important problem that they have to face [4,5,6,7,8,9,10]. Studies conducted by Picci et al. [11] have indicated that parents of children with NMDs have more anxiety and physical problems than those whose children are affected by other, more prevalent diseases. Besides, the physical, psychological and social quality of life (QoL) of the carer of a person with an NMD is also undermined by the overload they feel [12,13,14].

Many of the carers are vulnerable, since tackling a family member’s disease not only carries a physical and/or psychological burden, but also results in a series of economic costs derived from the disease, the degree of disability and its progression [15,16,17,18].

The economic coverage of the health system in Spain is high. People with NMD have access to almost every health service (except psychologists, physiotherapists, psychomotor educators, aquatic therapists, equine therapists and speech therapists). Furthermore, most of the drugs are covered by social security [19]. However, the low functional independence and the economic and social burden caused by NMD also makes it difficult for the health system to address them [20]. Likewise, a broad associative movement has been developed in Spain. For years, the organizations and federations of people with NMD in Spain have played an important role in the development of actions aimed at improving care and research in NMD [20].

The economic costs arising from informal care have been identified as one of the main factors to be faced by carers [17,18,21,22]. These costs are very high in NMDs [23,24].

Several studies divide the costs associated with NMD into four main categories: (a) direct medical costs (e.g., hospitalizations, visits to doctors or other health professionals, medical tests and evaluations, medication); (b) direct non-medical costs (e.g., disbursements related to non-medical aid); (c) costs associated with informal care (i.e., paid and unpaid informal care by the primary carer); (d) indirect costs (i.e., production losses for the patient and the primary carer due to absenteeism and reduced productivity during working time) [25,26,27,28,29].

Few studies include an assessment of indirect costs, making their estimation more difficult. Landfeldt et al. [27] indicated that 47% of the costs associated with the disease were derived from informal care and indirect costs, which represented a significant proportion of the total cost related to it [30].

Moreover, it has been observed that, while the economic burden increases in families of children with NMDs, QoL and psychological health decrease in these families [12,31,32,33].

When health expenditure increases, the economic calculations applied to health become particularly important. The nature of countries’ economies often means that economic support is provided for institutional care but not so much for home care, resulting in very few services being available free of charge. This directly affects families and, consequently, carers, who often have no choice but to provide informal home care [34,35].

The economic importance and the financial impact of NMDs in Europe have not yet been sufficiently investigated. According to Schepelmann et al. [36], most studies have focused on QoL rather than on economic data. Studies aimed at calculating the cost of a disease are useful for distributing resources, as they indicate which components have a greater role in the overall cost and make it possible to produce an economic evaluation of the possible interventions for primary, secondary and tertiary prevention [37,38]. The assessment of NMDs’ economic burden is very useful for setting intervention priorities and evaluating the effectiveness of therapeutic strategies [39]. It is important to implement these studies in order to reveal the real impact of a disease, which gives rise to a line of work to measure the loss of social welfare and move towards research for the assessment of health technology. Ultimately, their implementation would provide important information to the agents in charge of allocating health and social resources [40]. This study takes into consideration the needs described in previous research on carers and the absence of assessment of the economic impact that looking after children with NMDs has on carers’ psychological health. The purpose of this study is to evaluate the cost of informal care in the families of children with NMDs and the economic support provided by institutions. Additionally, how physical and psychological health (QoL, burden, satisfaction with life and somatic symptoms) is associated with sociodemographic variables, and how institutions provide economic support to these families.

## 2. Method

### Sample

A cross-sectional survey was carried out on carers of children with NMD. A total of 110 parents of children with NMDs participated in the study. A convenience sample of 110 people out of 200 who were contacted by associations and hospitals was used. Participants were recruited from two hospitals (Basurto University Hospital and Cruces University Hospital) and from the Spanish Federation of Neuromuscular Diseases.

The inclusion criteria were as follows: (a) being a parent of a child diagnosed with an NMD; (b) being over 18 years of age; (c) being a resident in Spain and having Spanish as one of their main languages; (d) having children under their care who were under 18 years of age.

The exclusion criteria were as follows: (a) being a parent of a child with any psychological or psychiatric diagnosis not secondary to an NMD; (b) presence of uncompensated sensory deficits that prevent the administration of the evaluation protocol; (c) illiteracy.

## 3. Measures

### 3.1. Sociodemographic Data

The first instrument was a 17-item ad hoc questionnaire, which collected the participants’ sociodemographic data (e.g., sex, age, academic level, type of employment and marital status).

### 3.2. Economic Data

The questionnaire of economic costs associated with care was included and had 248 items (technical aids the family had to buy, money spent in activities aimed at improving the health of the child and the carer, time spent in these activities and loss of work productivity). This questionnaire is supported by studies conducted with people with NMD [30,31,35,36,40].

### 3.3. Somatic Symptomatology

The PHQ-15 [41] was used to assess the somatic symptomatology associated with care. This questionnaire consists of 15 items referring to 15 possible physical problems that may have affected carers during the previous 4 weeks. Studies such as that by Ros, Comas and Garcia-Garcia [42] have demonstrated high internal consistency (Cronbach’s alpha 0.78). In the current study, the Cronbach’s alpha coefficient was 0.88.

### 3.4. Carer’s Overload

The Zarit Overload Scale [43] was used to assess the overload that participants may feel due to the caring experience. This assesses the workload of carers with dependents and determines the burden that the carer experiences in an overall score. It consists of 22 items, measured on a five-point Likert-type response scale. It has been shown to have a good internal consistency (Cronbach’s alpha 0.91) [44]. In the current study, the Cronbach’s alpha coefficient was 0.90.

### 3.5. Satisfaction with Life

The Satisfaction with Life Scale (SWLS) was employed to assess satisfaction in the life of the carer [45]. SWLS measures the individual’s level of satisfaction with life at that moment in time. It includes five questions and each question is rated on a 7-point scale (1 = not agree at all with the item, 7 = strongly agree). The possible range of this scale is from 1–7 per question. Cronbach’s alpha was 0.89 [46]. In the Spanish version, a high internal consistency of the scale has been demonstrated, with Cronbach’s alpha coefficients ranging from 0.79 to 0.89 [47]. In the current study, the Cronbach’s alpha coefficient was 0.88.

### 3.6. Quality of Life

QoL was assessed using the CarerQol [48]. This measures care-related QoL and consists of 7 items. The values of the CarerQol-7D ranged between 0.59 and 0.81. ICCs of the CarerQol7D had values between 0.55 and 0.94 [49]. Well-being (CarerQol-VAS) is measured in terms of happiness using an analog visual scale (VAS) with the “completely unhappy” and “completely happy” endpoints. Subjective burden (CarerQol-7D) is measured in seven dimensions: self-realization, relationship with the patient, mental health, economic problems, activities of daily living, external support and physical health [50]. The CarerQol-7D had a Cronbach’s alpha of 0.641 [51] in one study and a Cronbach’s alpha of 0.62 in another study [52]. In the current study, the Cronbach’s alpha coefficient was 0.63.

### 3.7. Level of Functional Independence

The Barthel Index [53] was used to assess the level of functional independence in personal activities of daily living. The internal consistency of the test ranged between 0.86 and 0.92 [54]. In the current study, the Cronbach’s alpha coefficient was 0.87.

## 4. Procedure

The researchers contacted the managers of hospitals and different NMD organizations in Spain by e-mail or phone to recruit potential participants. Two hospitals (the University Hospital of Basurto and the University Hospital of Cruces) and Spanish Federation of Neuromuscular Diseases agreed to recruit participants. After approval, all potential participants were sent an email that included the background information about the study, a consent form and a link to the survey. Surveys were sent to the participants with the assistance of Qualtrics. The protocol lasted approximately one hour (60 ± 20 min). Each participant signed an informed consent form. The research was approved by the Responsible Ethics Commission (Ref: ETK-39/18-19) and was conducted in accordance with the Declaration of Helsinki.

### Statistical Analysis

First, descriptive analyses were carried out on the annual direct social and health costs that the family (carer) of a child with an NMD had to bear. Second, the percentage of family income used to pay for direct costs was evaluated. To calculate the percentage of the family income that was used to cover each of the costs, the following equation formula was used: annual service cost-annual public funding received/income of the annual family unit.

Third, the replacement method was used to carry out cost analyses of informal care. The substitution or replacement method [55] was used, since it is a method widely used by researchers in Spain [56,57]. The technique involves assessing care hours spent by using the market price of a similar substitute service. In this case, the market price that would have to be paid for the service they provided free of charge was used as the valuation criterion. Therefore, this would be the expense in terms of formal care that society would have to pay in case the informal carer was unable to provide those services. The overall care hours were assessed on the basis of home carers’ hourly market wage rate, according to their collective agreement for the year 2018 [57]. Fourth, the loss of work productivity was analyzed by asking the participants directly.

Descriptive statistics were used to describe the participants. Continuous variables (e.g., sex or age) were described by mean and standard deviation, and categorical variables by frequency and percentage. The Kolmogorov–Smirnov test was used to test a normality of the outcome variables. Data were analyzed with the Mann–Whitney *U* test, Chi-Square and Kruskal–Wallis test. A *p* value of smaller than 0.05 was considered as statistically significant. IBM SPSS Statistics 26.0 was used for all analyses.

## 5. Results

One hundred and ten carers participated in this study: 91 women (82.8%) (average age 44.67 ± 7.25) and 19 men (17.2%) (average age 47.42 ± 11.07). Table 1 shows the data related to the sample distribution according to the type of NMD suffered by the affected child, marital status, academic level and employment status. At first, we carried out a study with carers of children with Duchenne muscular dystrophy (DMD), so this is why most children suffered DMD in this sample. The participants’ average years of education was 14.94 ± 6.91; their average number of children was 1.87 ± 0.97; the average age of the children with an NMD was 12.75 ± 6.56. Women represented the majority percentage of participants, and most of them had severely dependent children.

### 5.1. Social and Health Costs

It is relevant to note that all children go to a public health service, but parents also can go to a private health service, if they want. However, a public alternative does not exist in the case of physiotherapy. The highest annual of social and health expenses were the fees of a number of professionals; those were as follows: physiotherapists, external carers, speech therapists, private doctors, psychologists and aquatic therapies (see Table 2). The annual cost of technical aids was EUR 51,002.40 ± 102,974.38.

### 5.2. Physical and Mental Costs

Regarding the costs related to the physical and mental care of the carer, the greatest economic expense was associated with fees paid to visits with private doctors, fitness center fees, physiotherapy and psychological care, respectively (see Table 2).

### 5.3. Percentage of Family Income

A total of 97 people answered the question about the average monthly income per family unit. The average was EUR 3145.88 ± 3725.82 per month; the average income per year was EUR 37,750.63 ± 44,709.86.

Overall, the highest percentage of the household income was used to cover fees for physiotherapy, psychological care and speech therapy, with physiotherapy being the most dominant, respectively. The percentage of the family income spent on professionals for the physical and mental care of the carers of children with NMDs was to private doctors and physiotherapy, while the largest part of the income was used to cover private doctor fees (see Table 3).

### 5.4. Cost of Informal Care per Person with NMD

The replacement method was used to carry out cost analyses of informal care. The minimum hourly rate for live-in carers established by the Spanish Ministry of Employment, Migration and Social Security in 2018 was taken as reference. This rate was EUR 5.76 per hour. The average number of hours spent on the child’s care per week was 46.75 ± 31.33.

The average costs of informal care per person with NMD were established according to the level of disability and the differences between the three groups (mild, moderate and severe dependence) were statistically significant. The cost was higher in the case of children with an NMD who had severe disability (see Table 4).

### 5.5. Work Status

An evaluation was carried out of how many carers were working at the time of the survey, or if they had been working during the previous 12 months. Sixty-five carers either were working or had worked for the previous 12 months, and 45 were not working at the time or had not worked for the previous 12 months. Nine of those who worked before had to leave their jobs because of their child’s disease.

### 5.6. Inferential Statistics

The *Chi*-square test showed that fewer married carers left their employment than carers who lived alone, *χ*^2^(4) = 14.78, *p* = 0.005. The test also showed that those carers who did not go to a psychologist were less likely to leave their job than those who saw a psychologist, *χ*^2^(1) = 5.90, *p* = 0.023. There exists a relationship between the degree of dependence of the child and the carer leaving work, *χ*^2^(2) = 7.22, *p* = 0.027. Finally, those carers who had not visited a private doctor were less likely to leave their job than those who had visited a private doctor, *χ*^2^(1) = 7.07, *p* = 0.008.

The analysis of sociodemographic and clinical variables indicated differences between men and women in the somatic symptom score (*p* = 0.019); between the carer’s marital status and the carer’s overload scores (*p* = 0.019) and QoL (*p* = 0.012); between the occupation of the carer and the carer overload scores (*p* = 0.014), QoL (*p* = 0.007) and somatic symptoms (*p* = 0.013); between leaving employment and the carer’s overload (*p* = 0.032); between the use of psychological care for the carer and QoL (*p* = 0.003) and somatic symptoms (*p* = 0.001); between the use of physiotherapy services for the carer and QoL (*p* = 0.026), somatic symptoms (*p* = 0.022) and carer’s overload (*p* = 0.028) (see Table 5 and Table 6).

## 6. Discussion

The study results showed that the most important costs that caregivers of children with NMD must face is because of the non-existence of social protection and due to the child’s diagnosis. Moreover, this study found that carers need more public funding to ensure that they can have access to health services. Informal carers of NMDs spent higher number of hours than carers of people with other chronic disease [58]. On the other hand, these results indicate that carers of children with severe disability have higher levels of needs, and their increase is associated with economic costs. Our study also found that female, single or separated and unemployed carers showed worse physical and psychological health.

Study findings showed that informal care represents a major contribution to improving the QoL of people suffering from the disease; however, this type of care is not remunerated, even though for some carers, this implies them having to stop work or leisure time. While there has been an increase in the number of economic studies related to NMDs, there is still a need to carry out research on the economic calculations applied to healthcare in this area.

In the present study, 82.7% of the carers were female, which matched other studies [59,60,61,62,63]. A study offered a possible explanation for gender differences in coping with care and concluded that women can address problematic situations by offering emotional support and care, while men focus less on those concepts [64].

Our study describes a series of economic costs faced by carers of children with NMDs. The highest annual expenses were those spent on professional fees, namely, first, physiotherapy, followed by external carers, speech therapy, private doctors, psychological care and, finally, aquatic therapy. As to the costs incurred for the carer’s physical and mental care, the greatest economic expense was found to be related to private doctors, fitness centers, physiotherapy and psychological care. These results matched the study carried out in Spain with carers of children with DMD [58]. This study reported that all families incurred in monthly medical costs, and those costs were more than EUR 50/month. As stated before, some health services (such as psychologists, physiotherapists, psychomotor educators, aquatic therapists, equine therapists and speech therapists) are not covered by social security. Consequently, carers have to invest a considerable part of their salary in accessing these services. Analyzing the costs globally, the highest amounts were spent on technical aid for the child. This finding is consistent with the study by Larkindale et al. [65] on costs incurred by relatives of people with NMDs, where the highest costs were found to be related to technical aids aimed at improving the peoples’ mobility.

Professional carers’ costs spent by some participants in the study were lower than those found in children with Marfan syndrome [31], but higher in terms of non-medical costs (e.g., cost of informal care by family caregivers). Additionally, it is important to note that there is some public funding for those affected to ensure that they can have access to health services. However, funding from regional governments and non-profit organizations is virtually non-existent for expenses associated with professional support for carers. This can lead to social inequality for caregivers, produced by the environment itself and economic systems that are not able to meet their needs.

The number of average weekly hours spent by informal carers doing activities necessary for their child with an NMD was much higher than the number of hours spent by carers of children with other chronic diseases [66] and higher than the number of hours spent by carers of elderly people with disabilities [67]. Considering previous research conducted on carers of people affected by rare diseases (RDs), the number of average weekly hours was lower than that reported by the participants included in the present sample. This was mentioned in studies such as that by Hendriksz et al. [68], performed on people with Morquio syndrome, and that by López-Bastida et al. [13], carried out on carers of people with spinal muscular atrophy. In comparison with carers of people with amyotrophic lateral sclerosis, the average number of hours spent per week by carers was higher than that reported by the present sample [69].

Regarding the assessment of informal costs, the present study found that the higher the level of dependence of the child, the higher the costs. This is consistent with the study by Laskar, Gupta, Kumar, Sharma and Singh [70], which showed that as the demands of person with NMD care increased, costs increased accordingly. This is also supported by a study conducted in India on people with diseases that affected the locomotor system, which indicated that the financial burden was higher in families of children with severe dependence [70].

Overall, the highest percentage of the income of the family unit per year used to cover professionals’ fees was spent on professional care for the child. In particular, it was allocated to physiotherapy, psychological care and speech therapy, the former being the most dominant. The highest percentage of family income allocated to professional care services for the carers themselves was spent on private doctors, physiotherapy and psychological care. These percentages are important for families, particularly for the most disadvantaged ones, who are faced with a critical financial situation that affects all areas of their lives and increases their stress levels. The carers who were most affected were those with low income and those with children with high dependence [71].

The costs of informal care have been identified as one of the main factors driving the financial burden of the NMD, with expenses increasing as the disease progresses [17]. The replacement method has been used in this study, since it is a method widely used by researchers at national and international level [57,71,72,73,74,75,76].

Cost associated with care according to the substitution or replacement method was much higher than that of the present sample in comparison with studies carried out in the United States [73,77]. The cost associated with care according to the substitution or replacement method was much higher than that of the present sample. However, when factoring in the fact that the average hours were found to be lower than in this study, the carers’ salaries for the work done were higher. In comparison with a study carried out with carers of dependent people in the UK, the cost associated with care was also higher [72]. In an investigation carried out throughout Spain on carers of people with disabling diseases, the same method was used, but with different scenarios. As the study was performed in 2009, the salary of reference for that year was used, and therefore, the results are not comparable. It can be seen in different studies with carers of people with Alzheimer’s disease in the United States and in Spain that the annual informal costs are also higher than in the present study [75,76].

Nevertheless, the replacement method [55] shows a higher informal economic cost in this sample than in the study by Moreno and Guerrero [57], which studied multiple pathologies and included diseases with low and high prevalence. The study by Chevreul et al. [12] on carers of people with systemic sclerosis also showed lower informal costs than those in the present study. This result is similar when compared to that of Delgado et al. [56], which reported lower informal costs in carers of people with chronic heart failure. Another study on informal carers of people with dementia performed in the United States found that costs were lower than those of the present sample, considering similar scenarios using the replacement method [74].

This study also measured loss of work productivity, and it was seen that 58 percent of the carers either were working or had worked during the previous 12 months. However, of that 58 percent, our results indicated that eight percent of the carers had been compelled to leave their jobs, and those who were married were less likely to leave work. In couples, one of the members of the couple was able to take care of the child, and therefore, there were no high work absenteeism figures. This result may be explained by the fact that living alone often means that the father or mother is the main carer and consequently cannot spend much time doing other tasks [78]. It was also found that the use of psychological care services for the carers had an impact on them leaving their jobs. Carers who had not seen a psychologist had a lower dropout rate than those who had. One possible explanation is that their job could serve as a protective factor or an escape route to reduce the psychological consequences of the disease on the carer [79]. Finally, parents who had a child with moderate or severe disability had a higher rate of absenteeism than informal carers of children with mild disability or no dependence. It seems possible that this result is due to the fact that the dependence of the child, in many cases, requires specific care that is usually provided by the father or mother, and as the dependence increases, it requires more time, which may lead to one of the two parents leaving work. The use of medical services was also associated with greater rates of dropout from work, which could have been because of the increase in sick leave due to physical or psychological problems derived from the child’s illness.

One of the indirect consequences of the emotional effects of care and costs was the feeling of overload experienced by parents. Several psychological variables were analyzed. Differences between men and women were found in terms of somatic symptoms, with women obtaining the highest score. The literature has also indicated that female informal carers suffer more severe somatic symptoms than male informal carers [80,81]. Differences were found between the marital status of the carer, their burden and QoL. Those who were separated or single had higher scores in terms of feeling overloaded than the rest, and single people had lower QoL scores. This could be explained by the lack of social support perceived by those who did not have a stable partner [82]. This is consistent with other studies that have stated that the carer’s marital status can contribute to an excessive burden and consequently reduce QoL [83,84]. In addition, there were differences between the occupation of the carer and the overload scores (a greater overload was felt by those who were studying and did not work); QoL (retirees and students had poorer QoL scores); somatic symptoms (the unemployed had more severe somatic symptoms). The findings showed that all those who were not in a position to obtain paid work scored more highly in psychological distress measures. There were also differences between dropout from work and carer overload. Those who left work suffered from a stronger feeling of overload. Carer overload has been described in studies such as that by del Castillo et al. [85] as having higher scores in depression. Thus, a possible explanation to this relationship is that people who score highly in feeling overloaded may score highly in depression, and this has been associated with the probability of losing working hours [84,86].

The carers who used psychology services had higher scores in somatic symptoms, and those who used physiotherapy services also had lower QoL scores, higher scores in somatic symptoms and a stronger sense of feeling overloaded. This matched the study by Rodríguez-González et al. [87].

Study limitations included the length of the instruments and their duration, which might have influenced carers when deciding whether to complete it or not. Another limitation can be focused on the sample recruitment, with a major percentage of people affected by Duchenne muscular dystrophy. Besides, the groups created according to the level of dependency were not homogeneous. Other limitation of this study include the difficulty of making comparisons related to informal costs between international studies, as salaries are not homogeneous worldwide. Another limitation is related to the methodology of this study. This study is cross-sectional, and cause and effect cannot be inferred. Finally, sex ratio of participants is unbalanced, so the costs of caring for Spanish men cannot be inferred.

## 7. Conclusions

This study shows that carers of children with NMDs have to face a number of high costs related to their own physical and psychological health. Women represented the majority percentage of participants, and most of them had severely dependent children. Therefore, it was perceived that both the group of children with severe support needs, as well as their carers, had greater needs. In turn, it is important to note that there is some public funding for those affected to ensure that they can have access to health services. However, funding from regional governments and non-profit organizations is virtually non-existent for expenses associated with professional support for carers. Moreover, if these carers start from a low socioeconomic situation, dedicating themselves to caregiving increases their vulnerability. Therefore, these situations give rise to social inequality in this group. Finally, our study also found that carers who are female, single or separated and unemployed showed worse physical and psychological health.

Future lines of research could try to generate a unified protocol to be able to assess the informal costs of a disease both nationally and internationally in an equitable way. As previously mentioned, although research on costs incurred in connection with certain pathologies has increased, there is no methodology to assess them at present. Finally, a longitudinal study would be suitable in order to evaluate the evolution of costs attending the worsening of participants’ NMDs.

## Figures and Tables

**Table 1:** Distribution of socio-demographic characteristics (*n* = 110).

Socio-Demographic Variables		*n* (%)
Sex	Men	19 (17.2%)
	Women	91 (82.8%)
Marital status	Married	84 (76.4%)
	Living with their partner	7 (6.4%)
	Divorced	8 (7.3%)
	Separated	5 (4.5%)
	Single	6 (5.4%)
Academic level	Without school certificate	4 (3.6%)
	Compulsory education	11 (10%)
	Post-compulsory education	15 (13.6%)
	Intermediate-level vocational training	12 (10.9%)
	Higher-level vocational training	16 (14.5%)
	Ordinary degree	16 (14.5%)
	Bachelor’s degree (with honours)	23 (20.9%)
	Graduate	5 (4.5%)
	Master’s degree	2 (1.8%)
	PhD	6 (5.4%)
Employment status	Wage-earner	57 (51.8%)
	Unpaid work	3 (2.7%)
	Self-employed	7 (6.4%)
	Unemployed for health reasons	2 (1.8%)
	Unemployed for other reasons	15 (13.6%)
	Retired	5 (4.5%)
	Housework	12 (10.9%)
	Student	7 (6.4%)
	Disabled	2 (1.8%)
Type of NMD	Duchenne muscular dystrophy	40 (36.4%)
	Charcot–Marie–Tooth	11 (10%)
	Becker muscular dystrophy	5 (4.5%)
	Ullrich congenital muscular dystrophy	1 (0.9%)
	Spinal muscular atrophy	10 (9.1%)
	Myotonic dystrophy (Steinert disease)	12 (10.9%)
	Myopathies	7 (6.4%)
	Arthrogryposis multiplex congénita	1 (0.9%)
	Merosin-deficient congenital muscular dystrophy	2 (1.8%)
	Limb-girdle muscular dystrophy	5 (5.5%)
	Merosin-deficient congenital muscular dystrophy	3 (2.7%)
	Limb-girdle muscular dystrophy due to Fukutin-related protein (FKRP) deficiency	1 (0.9%)
	Corpus callosum hypoplasia	1 (0.9%)
	Hereditary spastic paraparesis	1 (0.9%)
	Congenital myasthenia	2 (1.8%)
	Collagen VI related muscular dystrophy	3 (2.7%)
	Sarcoglycanopathy	2 (1.8%)
	Pelizaeus Merzbacher disease	1 (0.9%)
	Friedreich ataxia	2 (1.8%)

**Table 2:** Annual direct social welfare and health-related cost (*n* = 110).

	n *	Annual Direct Cost (EUR) M ± SD	Annual Public Funding (EUR) M ± SD
Professionals required for the child			
Private doctor	39 (35.4%)	1032.5 ± 1686.5	23.44 ± 97.53
Psychological care	42 (38.1%)	992.06 ± 1549.79	21.25 ± 92.97
Physiotherapy	95 (86.3%)	1640.24 ± 1887.82	142.06 ± 680.71
External carers	18 (16.3%)	1189.82 ± 1632.19	0
Speech therapy	25 (22.7%)	1055.21 ± 633.49	148.18 ± 311.22
Psychomotor education	23 (20.9%)	405.47 ± 590.83	50.78 ± 215.18
Private tutors	24 (21.8%)	768.23 ± 602.91	78.26 ± 375.32
Learning support	11 (10.0%)	185 ± 381.01	0
Dietary supplements	47 (42.7%)	377.53 ± 484.32	0
Fitness centre	20 (18.1%)	424.2 ± 327.28	50 ± 223.6
Aquatic therapy	56 (50.9%)	859.93 ± 443.64	40.83 ± 260.34
Equine therapy	10 (9.0%)	727.78 ± 550.6	-
Total costs for professionals required for the child	110	1868.64 ± 2937.09	152.62 ± 457.92
Professionals required for the carer			
Psychological care	37 (33.6%)	305 ± 626.04	0
Physiotherapy	58 (52.7%)	344.89 ± 449.17	26.94 ± 139.77
Fitness centre	42 (38.1%)	346.96 ± 328.68	0
Private doctor	30 (27.2%)	644.5 ± 800.86	0
Support group	15 (13.6%)	32 ± 70.04	0
Training	63 (57.2%)	197.31 ± 485.73	0
Total costs for professionals required for the carer	110	306.80 ± 581.54	0

* Percentage of multiple responses: professionals selected/requested by carers.

**Table 3:** Annual percentage of annual income used to cover health-related costs by caregivers. (*n* = 110).

	n *	Annual Percentage (EUR) M ± SD
Professionals required for the child		
Private doctor	39 (35.4%)	2.68 ± 3.59
Psychological care	42 (38.1%)	4.37 ± 8.7
Physiotherapy	95 (86.3%)	4.44 ± 5.65
External carers	18 (16.3%)	3.5 ± 4.09
Speech therapy	25 (22.7%)	3.89 ± 3.54
Psychomotor education	23 (20.9%)	1.4 ± 1.97
Private tutors	24 (21.8%)	2.72 ± 2.8
Learning support	11 (10.0%)	1.02 ± 2.23
Dietary supplements	47 (42.7%)	2.16 ± 3.42
Fitness centre	20 (18.1%)	1.03± 1.16
Aquatic therapy	56 (50.9%)	1.78 ± 1.45
Equine therapy	10 (9.0%)	2.49 ± 2.49
Total annual percentage used to professionals required for the child	110	4.72 ± 10.15
Professionals required for the carer		
Psychological care	37 (33.6%)	1.02 ± 1.7
Physiotherapy	58 (52.7%)	1.21 ± 2.65
Fitness centre	42 (38.1%)	0.91 ± 0.74
Private doctor	30 (27.2%)	1.75 ± 2.78
Support group	15 (13.6%)	0.13 ± 0.25
Training	63 (57.2%)	0.86 ± 2.46
Total annual percentage used to professionals required for the carer	110	0.91 ± 2.11

* Percentage of multiple responses: professionals selected/requested by carers.

**Table 4:** Cost of informal care according to the level of disability. (*n* = 95).

Level of Disability	*n*	M ± SD
Mild dependence	33 (34.7%)	6563.84 ± 4254.00
Moderate dependence	14 (14.7%)	11,059.20 ± 5479.60
Severe dependence	48 (50.5%)	17,922.81 ± 8704.39

**Table 5:** Kruskal–Wallis test analysis of sociodemographic and clinical variables. (*n* = 110).

Clinical Variables	Sociodemographic		Mean Rank				
	Variables	*n*		Mean	*H*	*p*	*η^2^*
Carer’s overload					11.73	0.019	75
	Married	72	43.53	54.59			
	Living with their partner	6	48.92	56.5			
	Divorced						
	Separated	8	63.69	64.75			
	Single	4	72	69.25			
	Widowed	6	71.83	69.16			
		0	-	-			
Quality of life					12.84	0.012	0.084
	Married	73	43.71	12.36			
	Living with their partner	6	48.75	12.83			
	Divorced						
	Separated	8	69	15			
	Single	4	80.88	16.25			
	Widowed	5	59.5	13.6			
		0	-	-			
Carer’s overload					19.2	0.014	0.139
	Wage-earner	47	50.16	58.34			
	Self-employed	6	28.5	46			
	Unpaid work	3	14.83	39.66			
	Unemployed for health reasons	2	49.5	56			
	Unemployed for other reasons						
	Retired	13	54.58	60.23			
	Housework						
	Student	5	47.7	56			
	Disabled	12	35.21	49.16			
		6	76.75	72			
		2	76.5	73			
Quality of life					21.02	0.007	0.159
	Wage-earner	47	42.55	12.27			
	Self-employed	6	33.92	11.66			
	Unpaid work	3	33.5	11.33			
	Unemployed for health reasons	2	56.75	13.5			
	Unemployed for other reasons						
	Retired	13	66.04	14.38			
	Housework						
	Student	5	68.4	15			
	Disabled	12	37.63	11.83			
		6	72.17	15			
		2	76.75	15.5			
Somatic symptoms					19.42	0.013	0.159
	Wage-earner	41	37.55	10.31			
	Self-employed	5	25.3	7.4			
	Unpaid work	3	36.33	9.66			
	Unemployed for health reasons	2	58.75	17			
	Unemployed for other reasons						
	Retired	13	64.35	17.92			
	Housework						
	Student	5	44.8	11.8			
	Disabled	12	39.58	11			
		4	56.88	14.5			
		1	85.5	30			

**Table 6:** Mann–Whitney U test analysis of sociodemographic and clinical variables of the carers (*n* = 110).

Clinical Variables	Sociodemographic		Mean Rank				
	Variables	*n*		Mean	*U*	*p*	*R*
Somatic symptoms	Men	11	27	7.63	231	0.019	0.391
	Women	75	45.92	27			
Carer’s overload	Leave employment	8	38.5	68.75	96	0.032	0.41
	Not leave employment	46	25.59	55.91			
Quality of life	Use of psychological care	35	58.9	13.91	668.5	0.032	0.327
	Not use psychological care	60	41.64	12.13			
Somatic symptoms	Use of psychological care	29	55.81	15.44	440.5	0.022	0.379
	Not use psychological care	56	36.37	10.32			
Quality of life	Use of physiotherapy services	52	53.67	13.34	823	0.026	0.226
	Not use of physiotherapy services	43	41.14	12.11			
Somatic symptoms	Use of physiotherapy services	47	48.48	13.42	635.5	0.022	0.224
	Not use of physiotherapy services	38	36.22	10.39			
Carer’s overload	Use of physiotherapy services	53	53.53	60.18	820	0.028	0.235
	Not use of physiotherapy services	42	41.02	52.83			

## Data Availability

The datasets generated and/or analyzed during the current study are not publicly available, because they belong to the University of Deusto, but are available from the corresponding author (Alicia Aurora Rodríguez) on reasonable request.

## List of Abbreviations

NMDs: neuromuscular diseases; QoL: quality of life; RDs: rare diseases.
